# Digital Light Processing 3D-Printed Ceramic Metamaterials for Electromagnetic Wave Absorption

**DOI:** 10.1007/s40820-022-00865-x

**Published:** 2022-05-05

**Authors:** Rui Zhou, Yansong Wang, Ziyu Liu, Yongqiang Pang, Jianxin Chen, Jie Kong

**Affiliations:** 1 MOE Key Lab of Materials Physics and Chemistry in Extraordinary Conditions, Shaanxi Key Lab of Macromolecular Science and Technology, School of Chemistry and Chemical Engineering, Northwestern Polytechnical University, Xi’an, 710072 People’s Republic of China; 2 Key Laboratory of Optical System Advance Manufacturing Technology, Changchun Institute of Optics, Fine Mechanics and Physics, Chinese Academy of Sciences, Changchun, 130033 People’s Republic of China; 3 School of Electronic Science and Engineering, Xi’an Jiaotong University, Xi’an, 710049 People’s Republic of China

**Keywords:** Electromagnetic wave absorption, Metamaterial, Precursor-derived ceramics, 3D printing

## Abstract

Combining 3D printing with precursor-derived ceramic for fabricating electromagnetic (EM) wave-absorbing metamaterials has attracted great attention. This study presents a novel ultraviolet-curable polysiloxane precursor for digital light processing (DLP) 3D printing to fabricate ceramic parts with complex geometry, no cracks and linear shrinkage. Guiding with the principles of impedance matching, attenuation, and effective-medium theory, we design a cross-helix-array metamaterial model based on the complex permittivity constant of precursor-derived ceramics. The corresponding ceramic metamaterials can be successfully prepared by DLP printing and subsequent pyrolysis process, achieving a low reflection coefficient and a wide effective absorption bandwidth in the X-band even under high temperature. This is a general method that can be extended to other bands, which can be realized by merely adjusting the unit structure of metamaterials. This strategy provides a novel and effective avenue to achieve “target-design-fabricating” ceramic metamaterials, and it exposes the downstream applications of highly efficient and broad EM wave-absorbing materials and structures with great potential applications.

**Supplementary Information:**

The online version contains supplementary material available at 10.1007/s40820-022-00865-x.

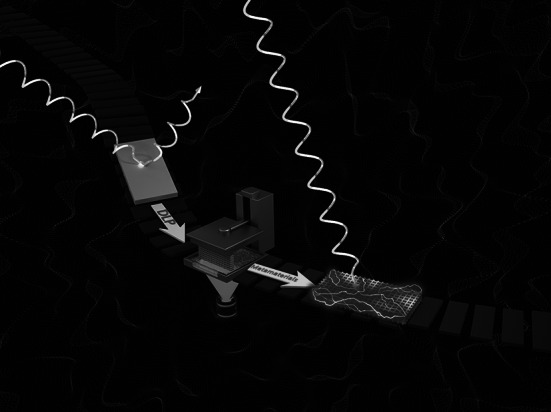

## Article Highlights

- A novel UV-curable polysiloxane precursor was synthesized with excellent UV curable performance and stability.
- The polysiloxane can fabricate Si–O–C ceramic with complex geometry via digital light processing 3D printing.
- The ceramic metamaterials show electromagnetic wave absorption with a low reflection coefficient and a wide effective absorption bandwidth even under high temperature.
- It provides a novel and effective avenue to achieve “target-design-fabricating” ceramic metamaterials with potential in EM wave-absorbing structures from nano to micro scale.

## Introduction

Electromagnetic (EM) wave-absorbing materials have rapidly gained attention owing to the accelerated development of the electronic industry and the increased environmental problems associated with EM wave interference and EM wave radiation [1–6]. Inorganic ceramics with tunable permittivity [7–9], high-temperature resistance [10–12], remarkable chemical durability, and excellent oxidation resistance [13–15] are potential candidates for EM wave-absorbing materials. However, their design and optimization with a low reflection coefficient (RC) and broadband effective absorption remain a great challenge [16–21]. The material’s components, micro/nanostructures, and metamaterial structures offer the possibility of tuning and achieving excellent EM wave absorption performance of ceramics [15, 20–23]. Particularly, the metamaterials originated approximately 20 years ago open a new opportunity for EM wave-absorbing materials by regulating the effective values of permittivity and permeability, achieving remarkable absorption performance in a specialized or wide frequency [24–29]. However, it is difficult for traditional ceramic processing, such as powder sintering, to process complex metamaterial structure of ceramic component [30, 31]. Therefore, it is highly desirable to develop a versatile and integrated method for advanced ceramic metamaterial EM wave-absorbing materials.

Precursor-derived ceramic (PDC) is a new representative forming processing for near-shape, complex structural, and multifunctional ceramics from small molecular or polymeric precursors [32–34]. As a potential dielectric-loss-type EM wave absorption material, PDC can adjust element components and crystalline domain structure via the design of precursor and control of the pyrolysis process. This can result in enhanced impedance matching and attenuation of EM wave-absorbing PDC materials [10, 32, 35–40]. The emergence of 3D printing technology for ceramics, such as digital light processing (DLP), overcomes the challenge of fabricating ceramics with arbitrarily complex shapes [41–47]. Therefore, the combination of DLP and PDC is a feasible strategy for the fabrication of high-performance EM wave-absorbing ceramics with complex micro/nanostructures and metamaterial structures in a rapid, mold-free, and inexpensive manner [41, 43, 48]. In a recent study [48], a modified polysilazane precursor using acrylate was developed, and several complex structures of Si–C–N ceramic/carbon nanotube composites were prepared using DLP 3D printing technology. Moreover, the combination and 3D printing technology with direct chemical vapor infiltration can fabricate Al_2_O_3_/SiC whisker EM wave-absorbing porous composites [23]. Nevertheless, presently, there are few studies on 3D printing EM wave absorption ceramic metamaterials. And to the best of our knowledge, there are no reports on the preparation of EM wave-absorbing ceramic materials using a combination of PDCs, metamaterial, and 3D printing technology.

Herein, a novel UV-curable and DLP-printable polysiloxane was designed as precursor for fabricating EM wave-absorbing ceramic metamaterials with complex geometric structures. By introducing double cross-linkable acrylic and silicon-vinyl groups, the polysiloxanes possess both excellent UV-curing performance and high ceramic yield. They also ensure linear shrinkage, no cracks, and a high density of the generated Si–O–C ceramic metamaterials after printing and pyrolysis. Owing to the high accuracy of the printed individual layer (25–100 μm), fast manufacturing speed (60 mm h^−1^), and large printing square (54 × 96 mm^2^), Si–O–C ceramic metamaterials with unique cross-helix structures can be achieved for EM wave absorption applications. The minimum reflection coefficient (*RC*_min_) reaches −36.33 dB (99.9% absorption), and the broad effective absorption bandwidth (EAB) is 3.76 GHz at the matching thickness of 2.90 mm in the X-band. This is a universal method that can be extended to Ku, K, Ka, and other bands, and it can be realized only by merely adjusting the unit structure.

## Experimental Section

### Materials

Dichloro(chloromethyl)methylsilane (DC(CM)MS) (> 98.0% purity) and photoinitiator diphenyl(2,4,6-trimethylbenzoyl)phosphine oxide (TPO) (97.0% purity) were purchased from Aladdin Bio-Chem Technology Co., Ltd. (Shanghai, China). Dichloromethylvinylsilane (DCMVS) (> 97.0% purity), 2-hydroxyethyl acrylate (HEA) (> 97% purity), and sodium hydride (NaH) were purchased from TCI Ltd. (Shanghai, China). Moreover, N, N-dimethylformamide (DMF) (99.8%, super dry with molecular sieves) and ethyl acetate (EA) were purchased from J.&K. Chemical Ltd. (Beijing, China).

### Synthesis of Polysiloxane (PSO)

The reaction was performed using the standard Schlenk technique. Under an argon atmosphere, 0.3 mol (50.05 g) of DC(CM)MS and 0.2 mol (29.08 g) of DCMVS were mixed in a 250 mL flame-dried flask equipped with a Teflon stir bar, septum, and high-vacuum stopcock. After the mixture was saturated with argon atmosphere under vigorous magnetic stirring, 0.5 mol (9.00 g) of deionized water was added dropwise within 30 min. After 1 h at ambient temperature, the mixture was heated to 75 °C and incubated for 4 h. Excess chlorodimethylvinylsilane (2 mL) was added as a capping agent. After completion of the reaction, 0.1 mol (12.03 g) of anhydrous magnesium sulfate was added to remove the residual moisture. Thereafter, the deposition was filtered. The clear filtrate was evaporated under a reduced pressure of 75 bar at 55 °C in the revolving distillation apparatus to remove the unreactive monomers and solvent. Therefore, PSO was obtained as a viscous transparent liquid with a yield of 78.7%.

### Synthesis of UV-Curable and DLP-Printable Polysiloxane

Dry DMF (200 mL) was added to a 500 mL flame-dried flask equipped with a Teflon stir bar, septum, and high-vacuum stopcock at 0 °C (ice-water bath) under an argon atmosphere. Thereafter, 0.36 mol (8.6 g) of NaH was added four times. After the NaH had dissolved, 0.3 mol (35.91 g) of HEA was added dropwise within 30 min. After the reaction was performed at 0 °C for 3 h, PSO (60 g) was added and heated to 70 °C for 8 h. The temperature of the mixture was reduced to 0 °C, and 5 mL of deionized water was added to quench the unreacted NaH. Thereafter, EA and the deionized water were used to extract and wash the product. The organic phase was evaporated under a reduced pressure of 75 bar at 55 °C in a revolving distillation apparatus to remove the residue solvent and EA. Finally, viscous transparent UV–PSO was obtained with a yield of 89.3%.

### DLP 3D Printing and Preparation of Ceramic Metamaterials

The DLP 3D printing of the designed ceramic metamaterial was conducted using an AUTOCERA-M DLP 3D printer (Beijing Ten Dimensions Technology Co., Ltd.) from a mixture of UV–PSO and photoinitiator TPO. The wavelength of the light source was 405 nm, and the irradiation intensity was 15 mW cm^–2^, as measured by LS125 Radiometer Photometer (Shenzhen Linshang Technology Co. Ltd). The exposure time was 6 s for a single layer. The shortest exposure time for a single layer was chosen when the curing strength met the stability of its own structure to obtain faster printing speed and manufacturing efficiency. The lifting speed of the supporting plant was 100 μm. The printing models were designed using the computer-aided design (CAD) software Materialize Magics V21 (Materialize Ltd., Leuven, Belgium). Subsequently, the resulting STL files were sliced for a 2D file output using 10 dim software (Beijing Ten Dimensions Technology Co., Ltd.) with a certain slicing thickness. After printing, the free-standing UV–PSO green bodies were moved into a UV-curing oven at 45 °C and were furtherly cured for 30 min (the irradiation intensity was 200 mW cm^−2^). Thereafter, the green body was transferred into a tube furnace (GSL-1700, Kejing New Mater. Ltd., China) under an argon stream. Thermal cross-linking was performed at 300 °C (heating rate: 3 K min^−1^; holding time, 2 h), followed by pyrolysis of approximately 1200 °C (heating rate, 3 K min^−1^; holding time, 3 h).

### Characterization and Measurements

Nuclear magnetic resonance (NMR) measurements were performed on a Bruker Avance 400 NMR spectrometer (Bruker BioSpin, Switzerland) to collect ^1^H and ^13^C spectra. The chemical shift was referenced to tetramethylsilane (TMS). Fourier transform infrared spectroscopy (FT-IR) spectra were recorded on a Nicolet Avatar 360 apparatus (Nicolet, Madison, WI, USA) with KBr plates and disks for liquid and solid samples, respectively. The UV-curing behavior of the UV–PSO was examined using differential photocalorimetry (DPC) (MDSC 2910, TA instruments, Newcoast, USA) and a UV source (L9566, Hamamatsu Photonics Co., Ltd. Beijing, China). The irradiation intensity of the UV source was 15 mW cm^−2^. The morphologies of the printed green body and ceramic metamaterial were characterized using a digital microscope system (DMS) (VHX-2000, KEYENCE) and scanning electron microscope (SEM) (Model 1530, LEO, Germany). Transmission electron microscopy (TEM, FEI Tecnai G2 F30) was performed at 200 kV, including electron diffraction analysis. For the preparation of the sample, a 5 μL droplet of an ultrasonically dispersed mixture of milled ceramic sample and alcohol (0.02 mg mL^−1^) was dropped onto a copper grid (200 mesh) coated with carbon film and dried at ambient temperature for 30 min. Other measurement details, such as size exclusion chromatography (SEC), thermogravimetric analysis (TGA), mass spectrometry (MS), powder X-ray diffraction (XRD), and imaging X-ray photoelectron spectroscopy (XPS), are presented in the Supplementary Information.

The complex permittivity ($\varepsilon = \varepsilon^{\prime} - j\varepsilon ^{\prime\prime}$) of the ceramic sample was measured using a vector network analyzer (VNA, MS4644A, Anritsu, Atsugi, Japan) using the waveguide method in the X-band. Based on the metal backplane, the reflection coefficient (RC) can be calculated using the measured relative complex permittivity and permeability based on the following equations [49–52]:

1 $$ {\text{RC}} = 20\,{\text{log}}_{10} \left| {\frac{{Z_{{{\text{in}}}} - 1}}{{Z_{{{\text{in}}}} + 1}}} \right| $$

2 $$ Z_{{{\text{in}}}} = \sqrt {\frac{{\mu_{r} }}{{\varepsilon_{r} }}} \,{\text{tan}}h\left[ {j\frac{2\pi fd}{c}\sqrt {\mu_{r} \varepsilon_{r} } } \right] $$

where *Z*_in_, *μ*_*r*_, and *ε*_*r*_ are the normalized input impedance, permittivity, and permeability of the materials, respectively. Moreover, *f*, *d*, and *c* represent the EM wave frequency, thickness (*m*), and velocity of the EM wave in the vacuum, respectively. By contrast, the *RC* of metamaterials at ambient temperature, 600 and 800 °C was tested on an arch-method reflectivity test system with a high-temperature heating oven using a vector network analyzer (VNC, N5230C, Agilent, USA). To ensure that the temperature was correct and the temperature distribution was uniform, the closed heating was adopted and the method of instant measurement was carried out after 10 min at the evaluated temperature. The dimensions of the metamaterial monolithic ceramic were 18 cm × 18 cm × 8 mm.

## Results and Discussion

### Design and Synthesis of UV-Curable and DLP-Printable Polysiloxane Precursors

**Fig. 1:**
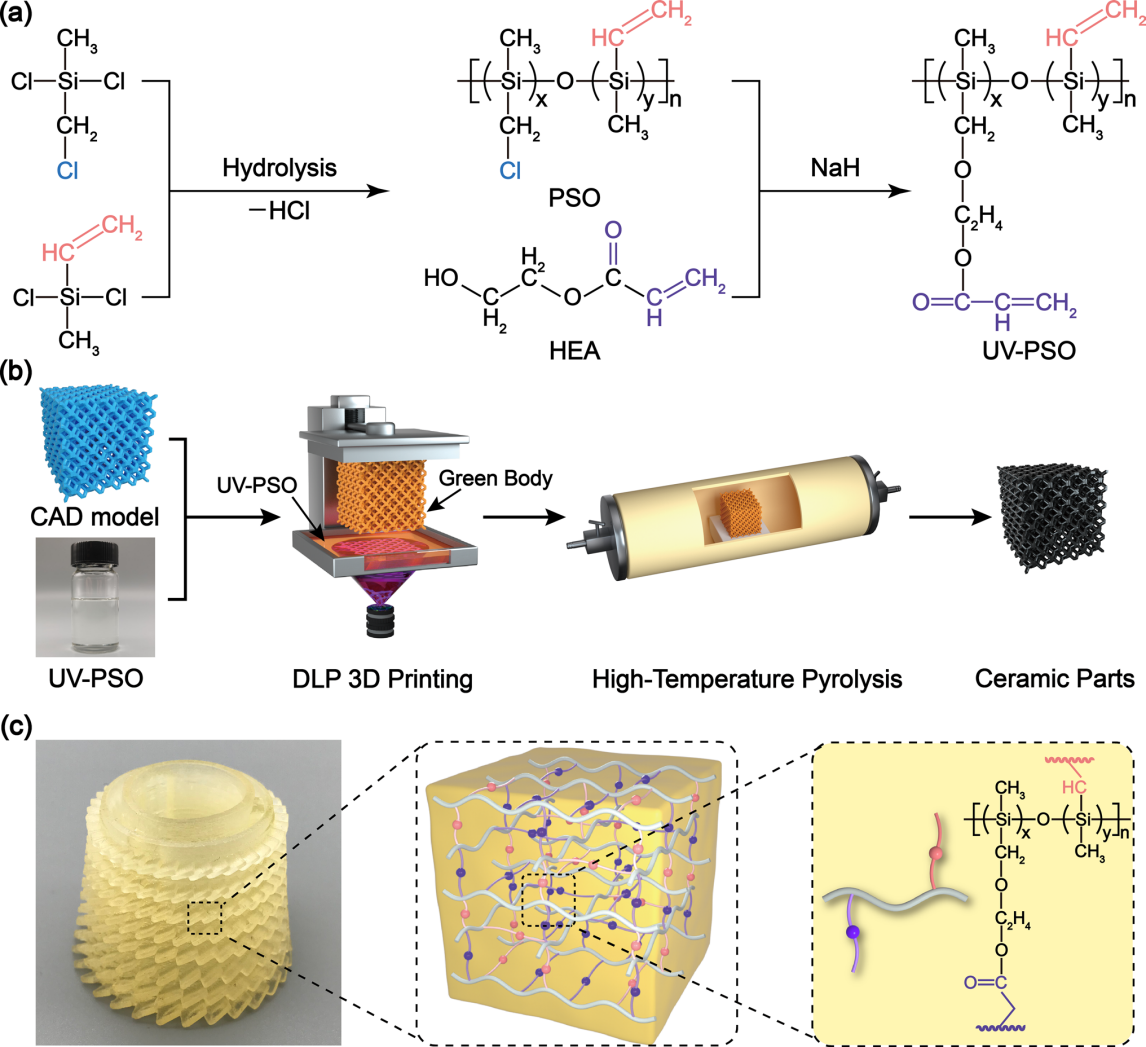
Synthesis route of the UV-curable polysiloxane and schematic DLP printing process and preparation of the designed Si–O–C ceramic metamaterial: **a** synthesis route of UV–PSO; **b** 3D printing of UV–PSO and pyrolysis process of Si–O–C ceramic metamaterial; and **c** photograph of a representative booster blade DLP-printed green body and two kinds cross-linked molecular structure of the UV–PSO

For the design of UV-curable and DLP-printable polysiloxane precursors, double cross-linkable groups, i.e., acrylic and silicon-vinyl groups were introduced from the monomers of HEA and DCVMS. As shown in Fig. 1, the polysiloxane (PSO) was synthesized via a hydrolysis and the condensation reaction between DC(CD)MS and DCVMS. Subsequently, UV–PSO, a viscous transparent liquid, was prepared through the etherification reaction between the –CH_2_–Cl side group of PSO and the hydroxyl of HEA with the assistance of NaH.

**Fig. 2:**
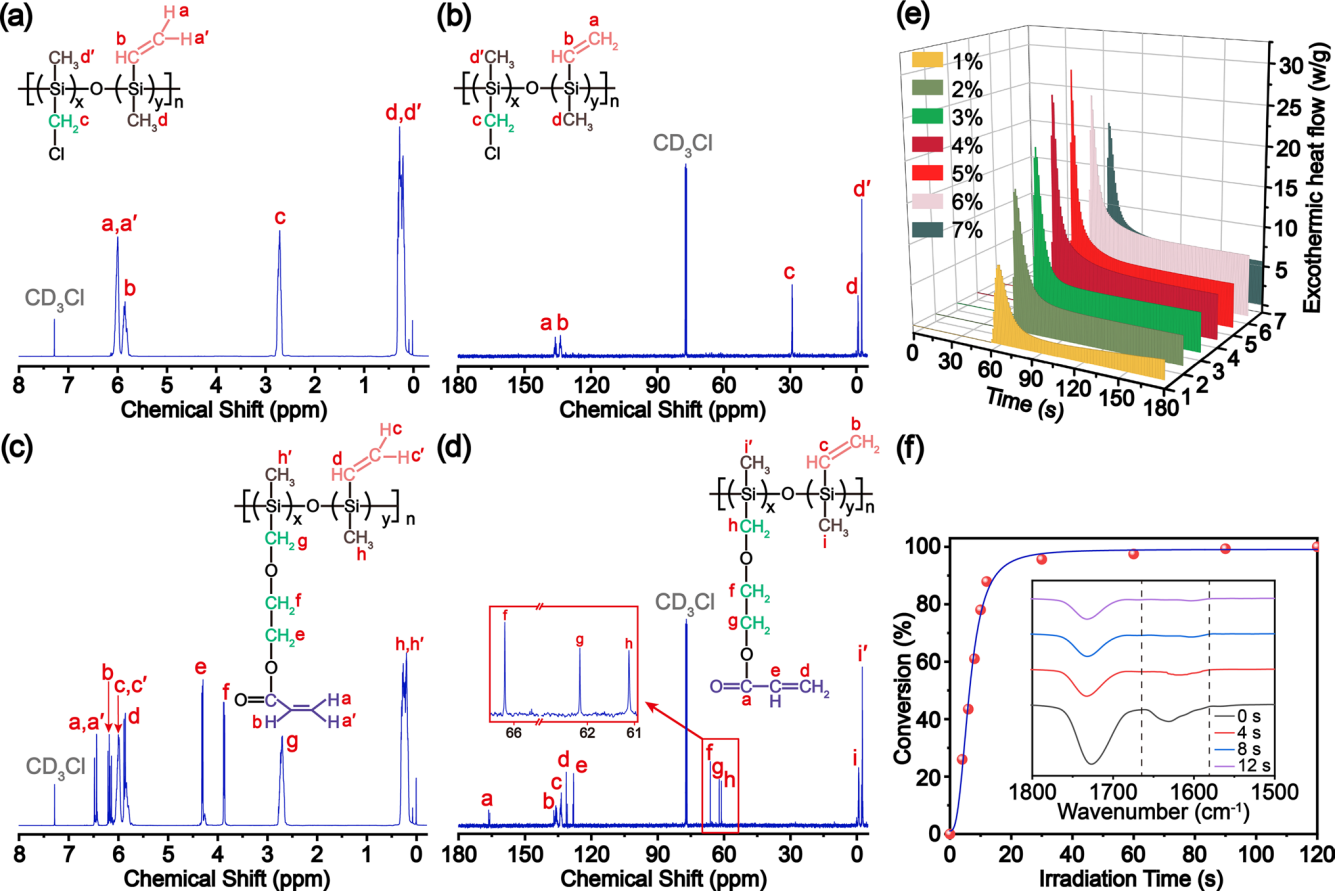
Structural characterization of the PSO and UV–PSO precursors: **a, c** ^1^H NMR spectra; **b, d** ^13^C NMR spectra; **e** DPC thermograms of UV–PSO with different photoinitiator concentrations; and **f** conversion percentage of acrylic group at different curing times (inset with FT-IR spectra)

The ^1^H and ^13^C NMR spectra of the PSO and UV–PSO are shown in Fig. 2. The ^1^H NMR spectrum reveals the existence of Si–CH_3_ bonds with a *δ* of 0–0.4 ppm and vinyl groups with a *δ* of 5.6–6.8 ppm. The characteristic signals at 2.75, 3.89, and 4.28 ppm were attributed to the methylene groups. The characteristic signals at 30.89 ppm (Si**C**H_2_Cl on DC(CM)MS, Fig. S1) remained in the same position in the PSO (Fig. 2b); nevertheless, it disappeared in the UV–PSO (Fig. 2d). The emerging characteristic signals at 66.30 ppm were attributed to the methylene group of UV–PSO (Si**C**H_2_OCH_2_**C**H_2_C(O)CHCH_2_). This change verifies the successful introduction of the acrylic group via the etherification reaction between the –CH_2_–Cl of PSO and the hydroxyl group of HEA. The clear assignment and matched integral value of all the protons illustrate the expected molecular structures of UV–PSO as shown in Fig. 1. The successful introduction of the acrylic group can also be confirmed by the characteristic C = O absorption at 1730 cm^−1^ in the FT-IR spectra (Fig. S2a). The number-average molecular weight (*M*_n_) was 1,110 g mol^−1^, and the polydispersity index (PDI) was 1.96 for the viscous transparent UV–PSO (Fig. S2b).

The photocuring reaction of UV–PSO is a radical polymerization reaction, so the addition amount of the initiator plays an important role on reaction rate. The “cage effect” caused by small addition amount and the “induction decomposition” caused by excessive addition amount led to a decrease in the initiation efficiency, so the UV-curing behavior of UV–PSO was studied before printing. The UV-curing behavior of UV–PSO was studied via differential photocalorimetry (DPC). The UV–PSO and various mass fractions (2–6 wt%) of TPO were mixed. The photoinitiator can decompose into free radicals under the UV irradiation and can initiate cross-linking of the acrylic group. The obvious exothermic heat flow in the DPC thermograms (Fig. 2e) verified the cross-linking reaction of the mixture exposed to 15 mW cm^–2^ under UV irradiation. The heat flow rapidly increased, with a sharp exothermic peak after the UV exposure. The maximum value of the heat flow was 28.89 W g^−1^ for the mixture with 5 wt% TPO, indicating that 5 wt% is the best amount. There are two types of unsaturated double bonds (silicon-vinyl and acrylic groups) in the UV–PSO. The silicon-vinyl double bond cannot be polymerized by TPO in a short time (several seconds) because of its low activity, so the conversion percentage (CP) of acrylic double bond can represent the photopolymerization performance of UV–PSO. The CP of the unsaturated bonds belonging to the acrylic group of the UV–PSO during the UV curing was determined using FT-IR. The carbonyl peak at 1730 cm^−1^ was used as the internal standard for normalization. The complete reaction of the acrylic group was achieved after 2 min based on UV irradiation, which was confirmed by the FT-IR results. At this time, the unsaturated double bond peak at 1637 cm^−1^ only belonged to the silicon-vinyl group, and the peak area was marked as A_*e*_. The peak area of the unsaturated double bonds at 1637 cm^−1^ at any time is recorded as A_*t*_, and that of the carbonyl bonds at 1730 cm^−1^ at any time is recorded as B_*t*_. The CP of the acrylic double bond can be calculated as follows [53]:

3 $$ {\text{CP}} = \left( {\frac{{\frac{{A_{0} }}{{B_{0} }} - \frac{{A_{t} }}{{B_{t} }}}}{{\frac{{A_{0} }}{{B_{0} }} - \frac{{A_{e} }}{{B_{e} }}}}} \right) \times 100\% . $$

Considering Fig. S3, the peak at 1637 cm^−1^ decreased rapidly with an increase in the irradiation time. This indicates that the cross-linking reaction between the unsaturated bonds occurred rapidly. As shown in Fig. 2f, the CP reached 61.03% after 8 s and finally reached 95.62% at 30 s. The UV–PSO is extremely efficient in photopolymerization owing to the high reactivity of the unsaturated bond covalent with the carbonyl group. The above results demonstrate that the UV–PSO can be rapidly cured under UV irradiation, which is critical for the subsequent DLP printing.

### DLP Printing and Ceramic Formation of Polysiloxane Precursors

**Fig. 3:**
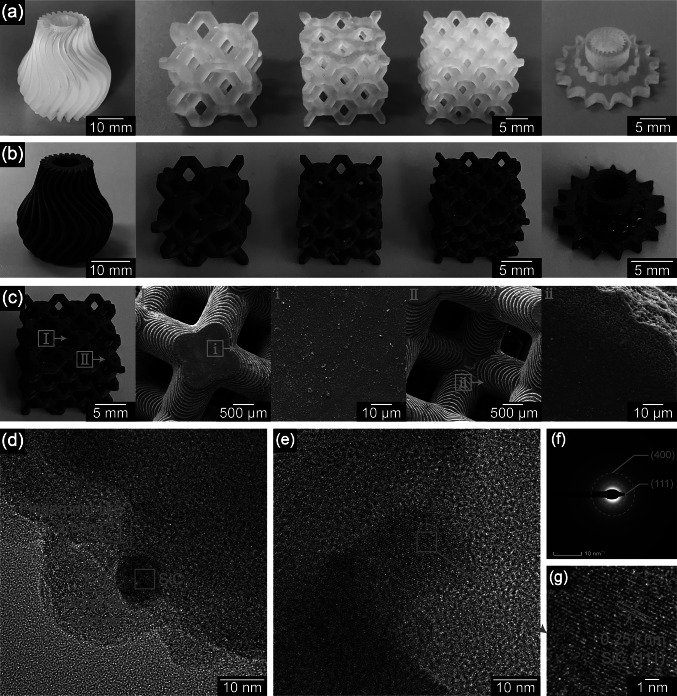
Morphology and structures of 3D-printed green bodies and the corresponding pyrolytic ceramics: **a** typical structure of green bodies prepared by the DLP 3D printing; **b** corresponding structure of Si–O–C ceramic parts; **c** surface morphology SEM images of the ceramic cell structure; **d, e** TEM images; **f** selected area electron diffraction (SAED) image; and **g** lattice fringe of SiC

As shown in Fig. 1b, the 3D printing was performed using a DLP printer with a light wavelength of 405 nm and an irradiation intensity of 15mW cm^−2^. DLP-printable polysiloxane precursors are suitable for various structural models as shown in Fig. 3a. The acrylic group in the UV–PSO endows the polymer with rapid cross-linking property to fix the shape in few seconds in the printing process. The silicon-vinyl group provides secondary thermal cross-linking that can further increase the cross-linking density and yield of ceramics. Therefore, these DLP-printed structures show excellent adaptability to the intrinsic properties of the material of various special structures and printing accuracies. Considering the SEM images of the green body in Fig. S4, the layer-by-layer fabrication was observed, and the thickness of each layer was ~ 100 μm (equal to the set layer thickness). Furthermore, the DLP-printed structures can be well maintained owing to the high CP during the printing and ceramic yield of 43.74%, even after pyrolysis at 1200 °C in an argon atmosphere (Fig. 3b). The TG–MS test and analysis results are shown in Figs. S5–S6. The ceramic parts retained their original shape, and the linear shrinkage rate was 34.7% (Calculated by measuring the printed green bodies and pyrolytic ceramics). Because the shrinkage is isotropic, it does not result in any deformation of the pyrolyzed structure. Considering the Si–O–C ceramic parts in Fig. S4, the layer structures were still intact, and the thickness of each layer was ~ 65 μm, which corresponds to the shrinkage rate of the entire ceramic parts. The pyrolyzed Si–O–C ceramic parts have a high density of 1.84 g cm^–3^ (tested by the Archimedes drainage method).

Figure 3c shows the surface morphology of the ceramic structure at different magnifications. The layer-by-layer fabrication and dense surface can be observed (pores only appeared in the range of 10 μm at the edge). The TEM images in Fig. 3d–g reveal the onset of crystallization with scattered *β*-SiC crystals inside the amorphous matrix. The bright-field images show small crystallites of few nanometers in the Si–O–C region. High-resolution imaging can identify crystallites as graphite and *β*-SiC based on the lattice spacing and diffraction pattern. The small crystals (5–10 nm) and the high fraction of the remaining amorphous matrix indicate that crystallization had just begun. The element mappings of Si, O, and C distributed in different colors are shown in Fig. S7. XPS analyses in Fig. S8 prove the elemental mappings of the existence of Si, O, and C elements in the ceramics. The representative atomic composition and formula of the ceramic were Si_1_O_3.51_C_9.22_. The main C 1* s* peak was at 284.8 eV (81.81%), which was detected along with the graphitic carbon. The Si 2*p* was deconvoluted into Si–O (83.66%) and Si–C (16.34%) peaks. The excess oxygen content mainly resulted from the O_2_ absorption of the ceramic powder before the XPS measurements. Furthermore, FT-IR and XRD in Fig. S9 illustrate that after pyrolysis, most regions of the ceramic parts were amorphous silica and carbon, and some β-SiC crystals were present in them.

### DLP-Printed Ceramic Metamaterials for Electromagnetic Wave Absorption

Impedance matching and attenuation are key to EM wave absorption performance [54, 55]. They are closely related to the complex permittivity, multi-scale structure, and thickness of the material [56–58]. Based on impedance matching, attenuation, and effective-medium theory [55, 59–61], various adjustments of the complex permittivity of the ceramic metamaterials were realized, and they further improved the EM wave-absorbing property through the design of microstructure and macrostructure.

**Fig. 4:**
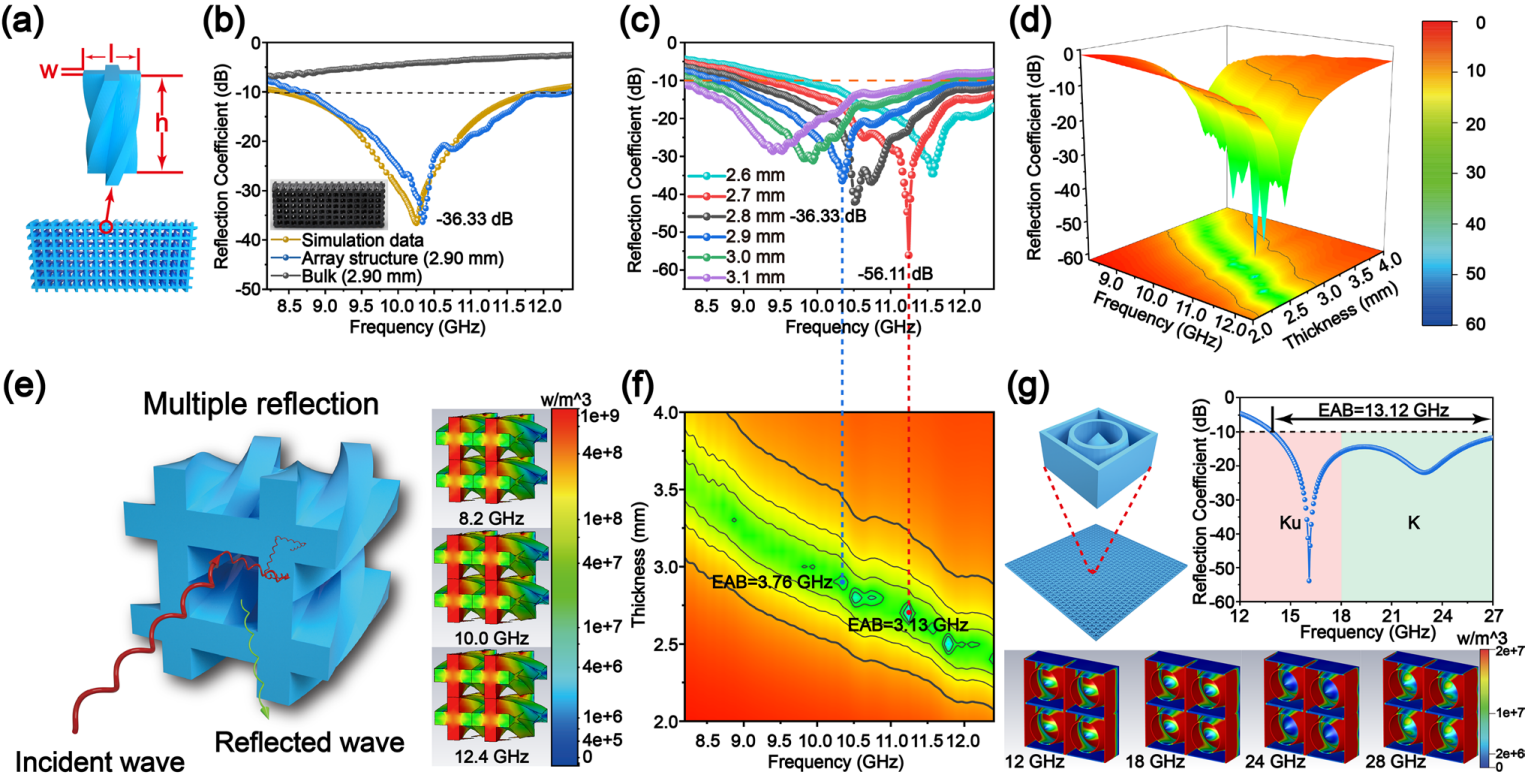
Design and fabrication of Si–O–C ceramic metamaterial structure in X-band: **a** unit and array structure models; **b** calculated RC values of the bulk sample, array structure sample, and the simulated data of the array structure; **c** RC versus frequency and thickness corresponding to **d** 3D graph and **f** 2D projection diagram; **e** multiple reflection mechanism; and **g** metamaterials structure design suitable for Ku and K bands

Considering Figs. 4a and S10, based on the complex permittivity in the X-band of the DLP-printed Si–O–C bulk ceramic without EM wave absorption, we designed a cross-helix array structure using the electromagnetic field simulation software (CST Studio Suite 2019, Dassault Systemes, Paris, France). The parameters (*w, l, h*) of the unit structure (*w* = 0.45 mm, *l* = 1.43 mm, *h* = 2.90 mm) with the optimization objective of the simulation calculation RC (≤ –10 dB) of array structure were optimized. Based on the cross-helix array structure model, the corresponding ceramic metamaterials in Fig. 4b were successfully prepared via DLP printing and subsequent 1200 °C pyrolysis of the UV–PSO. Considering Fig. S10a–b, the real (*ε*′) and imaginary (*ε*″) parts of the complex permittivity of the bulk ceramic were in the ranges of 21.79–24.32 and 9.71–9.01, respectively. Nonetheless, regarding the cross-helix array structure ceramic, the range was 7.24–6.75 and 3.95–3.23, respectively. It can be observed that ε′ and ε″ of the array structure were significantly reduced, which is conducive for impedance matching. Furthermore, the loss tangent value was used to represent the attenuation of the material. As shown in Fig. S10c, compared to the bulk sample, the loss tangent value of the array structure sample was only slightly reduced, indicating that the array structure had no significant impact on the attenuation of the material while significantly reducing the complex permittivity.

However, a good impedance matching is the premise of the EM wave-absorbing properties of materials, which determines the transmission behavior of EM waves in materials. An imbalance in the impedance matching will lead to a strong reflection of the EM wave at the interface of the material. Impedance matching characteristics can be evaluated using $\left|{Z}_{\mathrm{in}}-1\right|$. The ideal impedance matching condition $\left|{Z}_{\mathrm{in}}-1\right|$ tends to zero. Almost all the EM waves incident on the material surface were absorbed, and there was no reflection phenomenon. Considering Fig. S10d, the $\left|{Z}_{\mathrm{in}}-\left.1\right|\right.$ of the array structure sample (2.90 mm) was far less than that of the bulk sample (2.90 mm). This indicated that the array structure had a better impedance matching, which was achieved from the regulation of complex permittivity of equivalent medium in a reasonable range. Figure 4b shows a comparison of the measured RC data of the bulk and array samples and the simulation data of the array structure. The bulk sample had no absorption properties in the X-band, whereas the cross-helix array structure showed excellent EM wave-absorbing performance. Moreover, the RC value of the array structure was highly consistent with the corresponding simulated data, indicating the correctness of the simulated calculation. Figure 4c, d, and f shows the 2D curve, 3D graph, and 2D projection diagram of the calculated values of the RC for different thicknesses of the cross-helix array structure sample in 8.2–12.4 GHz. When the thickness was 2.7 mm, the *RC*_min_ was −56.11 dB, and the EAB was 3.13 GHz (9.27–12.4 GHz). When the thickness of the sample increased by 2.90 mm, the EAB was further increased to 3.76 GHz.

Although the bulk sample did not possess EM wave absorption, after the cross-helix array design, the ceramic metamaterial showed excellent absorption performance. This can be attributed to the following three aspects. First, the cross-helix array structure showed excellent impedance matching with free space. Considering Fig. S10d, the $\left| {Z_{{{\text{in}}}} - \left. 1 \right| } \right.$ of the bulk ceramic was greater than 0.8 in the X-band, resulting in a large number of EM waves reflected on the surface. The $\left| {Z_{{{\text{in}}}} - \left. 1 \right|} \right.$ of the metamaterial was less than 0.25, and even close to zero at a specialized frequency, and the ideal impedance matching significantly reduced the reflection of the EM wave. Second, regarding Fig. 4e, when the EM wave was incident on the surface of the metamaterial, the spiral pore-like structure caused multiple reflections and absorptions of the EM waves. Third, considering Fig. S10c, the high dielectronic loss performance of the Si–O–C ceramic itself was helpful for increasing the absorption performance. The *sp*^2^ hybrid carbons in the ceramic can improve the movement ability of the electrons. Owing to the action of an electromagnetic field, the electron movement lag in the dipole could cause additional polarization relaxation, which would lead to an increase in the dielectric constant and loss tangent. Crystalline carbons (graphitic carbon and fingerprint-like carbon) and silicon carbide crystals would form a nanocrystalline boundary between the nanocrystalline and amorphous phases. When an electromagnetic wave is incident on the material, the charge accumulated on the non-uniform interface causes interface polarization and relaxation, further enhancing the dielectric loss performance. Defects in in situ crystalline carbons can be used as tiny dipoles or polarization centers, leading to defect polarization, polarization relaxation, and microwave energy dissipation.

**Fig. 5:**
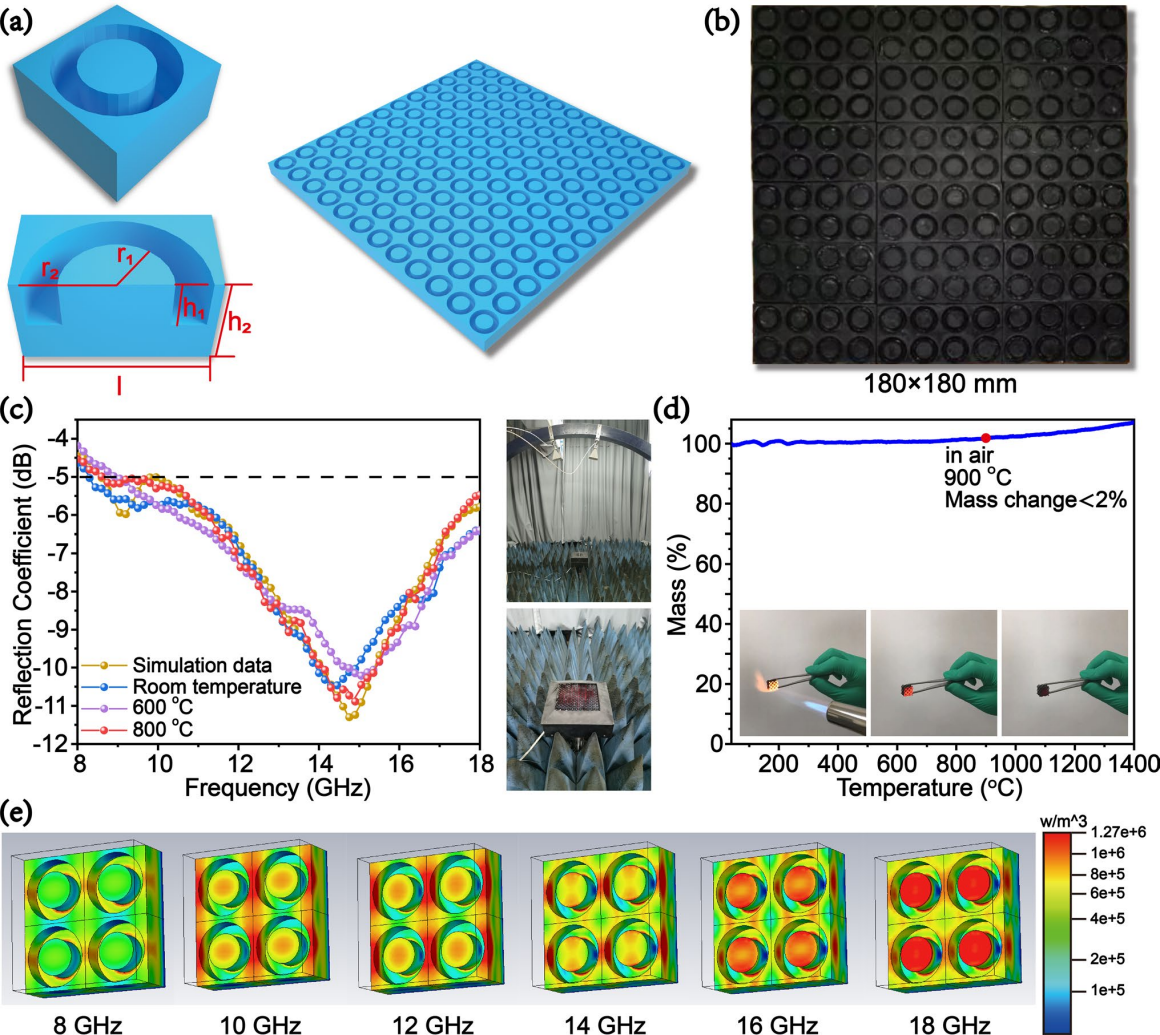
Design and fabrication of Si–O–C ceramic metamaterial structure for practical application in X-Ku band: **a** unit and array structure models; **b** actual photograph of the sample; **c** comparison of the measured RC data at different temperatures and simulation data; **d** high-temperature oxidation resistance of Si–O–C ceramic parts; and **e** energy flow density of the simulated array structure at different frequencies

To verify the feasibility of the practical application of the DLP 3D printing ceramic metamaterials for the EM wave absorption at high temperatures, we designed and prepared ceramic metamaterials with a simple structure that was easy to prepare in a larger scale and efficient based on the industrial application standard (RC < –5 dB). The optimized unit structure is shown in Fig. 5a, where *r*_*1*_ = 4.08 mm, *r*_*2*_ = 6.74 mm, *h*_*1*_ = 4.42 mm, *h*_*2*_ = 8.00 mm, and *l* = 15.00 mm. Limited by the size of the support plant of the printer, we printed and pyrolyzed 18 pieces (30 × 60 mm^2^ as shown in Fig. S11) and spliced them to obtain the test sample for the arch reflectivity test (Fig. 5b). The *RC* was tested at room temperature (25 °C), 600 °C, and 800 °C as shown in Fig. 5c. The *RC* of the DLP-printed ceramic metamaterial was highly consistent with the simulation data. The EM wave absorption performance showed a high stability with an increase in temperature, which is attributed to the excellent high-temperature oxidation resistance of Si–O–C ceramics (as shown in Fig. 5d). The high-temperature oxidation resistance of Si–O–C ceramic parts is due to the SiO_2_ passive oxide layer formed in the ceramics, which hinders the diffusion of oxygen to control oxidation. At 800 °C, the RC_min_ was −10.89 dB with an EAB of 9.35 GHz (8.65–18.00 GHz). The energy flow density (representing the intensity of dielectric loss) of the simulated array structure at different frequency points (Fig. 5e) illustrates that the structural design had a significant regulatory effect on the EM wave absorption performance. The above results show that the structural designs can control the EM wave-absorbing properties of materials. Furthermore, this strategy is generally applicable for several types of EM wave-absorbing metamaterials at different frequencies. For example, we can design other ceramic metamaterial structures for EM wave absorption in the X (8.2–12.4 GHz), Ku (12.0–18.0 GHz), K (18.0–27.0 GHz), and even Ka (27.0–40.0 GHz) band (Figs. 4g, S12). The DLP 3D printing of this precursor with high precision (individual layer, 25–100 μm) can achieve the manufacturing of those metamaterial structure. The theoretical calculation of the RC curves shows that design, and optimization of the metamaterial can achieve a wide range adjustment of the equivalent complex permittivity and the absorbing performance of the equivalent medium. It can provide theoretical support for the development of high-efficiency broadband EM wave-absorbing metamaterials.

## Conclusion

We developed a novel UV-curable polysiloxane precursor that could be used in DLP 3D printing to fabricate ceramic parts with complex geometric structures. The UV–PSO was synthetically simple, inexpensive, and stable with long-term storage. The double cross-linkable acrylic and silicon-vinyl groups provide the precursor with high-efficiency UV-curing properties and high ceramic yield simultaneously. The manufacturing of various Si–O–C ceramic structures was achieved with a high accuracy of the printed individual layers, fast manufacturing speed, and large scale. The ceramic parts pyrolyzed from the DLP 3D-printed green bodies with complex geometric structures and high precision (individual layer, 25–100 μm) can retain their original shape with no cracks, high density, linear shrinkage, and high ceramic yield without any fillers. Furthermore, the cross-helix array Si–O–C ceramic metamaterials can be fabricated via the DLP 3D printing technique for EM wave absorption. Benefiting from the designable structure and effective synergy, the 3D-printed Si–O–C ceramic cross-helix array structure showed an optimal EM wave absorption performance. This is a universal method that can be extended to other bands, and it can be realized by merely adjusting the unit structure. This study provides a new method with high design freedom and efficiency to manufacture tunable EM wave-absorbing ceramic materials or metamaterials with a wide EAB by combining PDC and 3D printing technology.

## Supplementary Information

Below is the link to the electronic supplementary material.

Supplementary file1 (PDF 1468 kb)
