# Supplementary material for: Digital Light Processing 3D-Printed Ceramic Metamaterials for Electromagnetic Wave Absorption
Source: Nanomicro Lett. 2022 May 5;14:122. doi: 10.1007/s40820-022-00865-x (PMC9072614; doi:10.1007/s40820-022-00865-x)
Supplement: Supplementary file 1 — Supplementary file1 (PDF 1468 kb) [file 40820_2022_865_MOESM1_ESM.pdf]

Supplementary Information for

Digital Light Processing 3D–printed Ceramic Metamaterials for Electromagnetic Wave Absorption

RuiZhou[1], Yansong Wang[2], Ziyu Liu[1], Yongqiang Pang[3], Jianxin Chen[1], Jie Kong[1,] *

[1]MOE Key Lab of Materials Physics and Chemistry in Extraordinary Conditions, Shaanxi Key Lab of Macromolecular Science and Technology, School of Chemistry and Chemical Engineering, Northwestern Polytechnical University, Xi'an 710072, P. R. China

[2]Key Laboratory of Optical System Advance Manufacturing Technology, Changchun Institute of Optics, Fine Mechanics and Physics, Chinese Academy of Sciences, Changchun 130033, P. R. China

[3]School of Electronic Science and Engineering, Xi'an Jiaotong University, Xi'an 710049, P. R. China

*Corresponding author. E–mail: kongjie@nwpu.edu.cn (Jie Kong)

S1 Experimental Section

Size exclusion chromatography (SEC) measurement was performed on a system equipped with a Waters 515 pump, an autosampler and two MZ gel columns (10^3 and 10^4 Å) with a flow rate of 0.5 mL min^{-1} in THF (HPLC grade) at 25 °C. The detectors included a differential refractometer (Optilab rEX, Wyatt) and a multiangle light scattering detector (MALS, Wyatt) equipped with a 632.8 nm He−Ne laser (DAWN EOS, Wyatt). The refractive index increments of the polymers in THF were measured at 25 °C using an Optilab rEX differential refractometer.

Thermogravimetric analysis (TGA) and mass spectrometry (MS) analysis was performed on a simultaneous thermal device (STA, 449C Jupiter, Netzsch, Gerätebau GmbH, Selb, Germany) coupled with a quadrupole mass spectrometer (QMS, 403C Aëolos, Netzsch, Germany). The measurement was done under a steady flow of argon (40 ml/min), with a heating rate of 10 K/min at a range from ambient temperature to 1400 °C.

Powder X−ray diffraction (XRD) measurement was conducted on an X'Pert Pro Pro Powder diffractometer from PANalytical (Cu Kα radiation, 40 kV, 40mA) (D/Max2550VB+/PC). The X'Celerator Scientific RTMS detection unit was used for detection.

Imaging X−ray photoelectron spectroscopy (XPS) measurements were conducted on a K−Alpha spectrometer (Axis Ultra, Kratos Analytical Ltd., U.K.) and the core level spectra were measured using a monochromatic Al Kα X−ray source (hν = 1486.7 eV). The analyzer was operated at 23.5 eV pass energy and the analyzed area was 200−800 μm in diameter. The lowest energy resolution is 0.48 S−3 eV (Ag $3d_{5/2}$). Binding energy was referenced to the adventitious hydrocarbon C 1s line at 284.8 eV. The curve fitting of the XPS spectra was performed using the least−squares method.

S2 Supplementary Figures

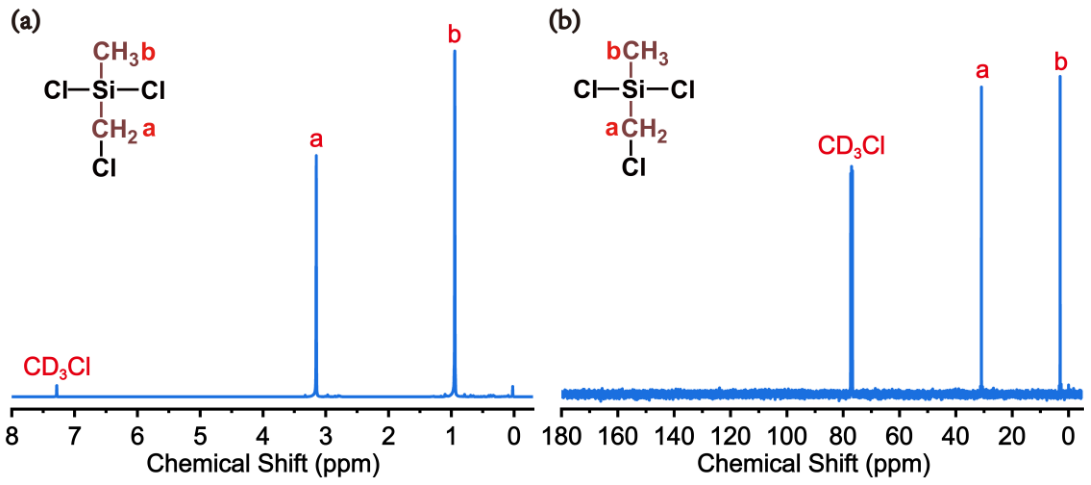

Fig. S1 (**a**) ^{1}H NMR spectrum and (**b**) ^{13}C NMR spectrum of Dichloro(chloromethyl)methylsilane

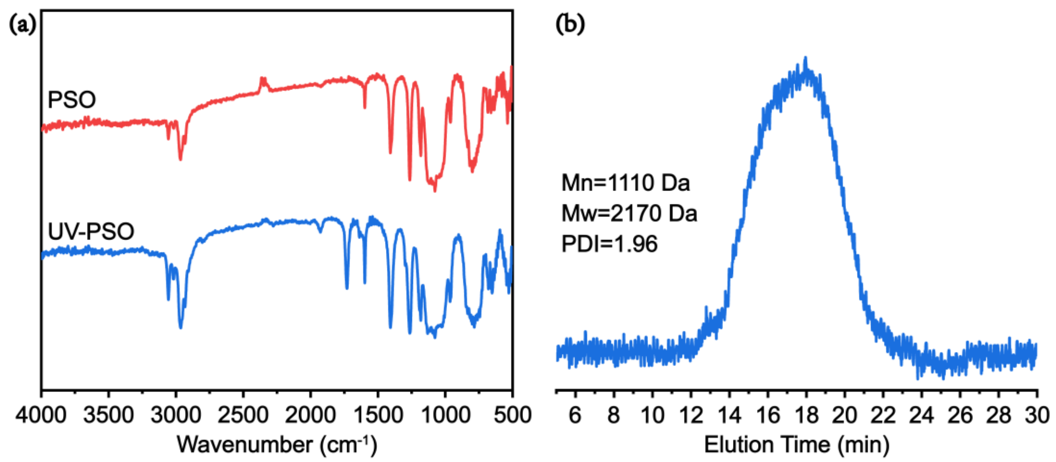

Fig. S2 (**a**) FT−IR spectra of PSO and UV−PSO, (**b**) SEC trance of UV−PSO at a flow rate of 0.5 mL min^{-1} in THF at 25 ^{o}C

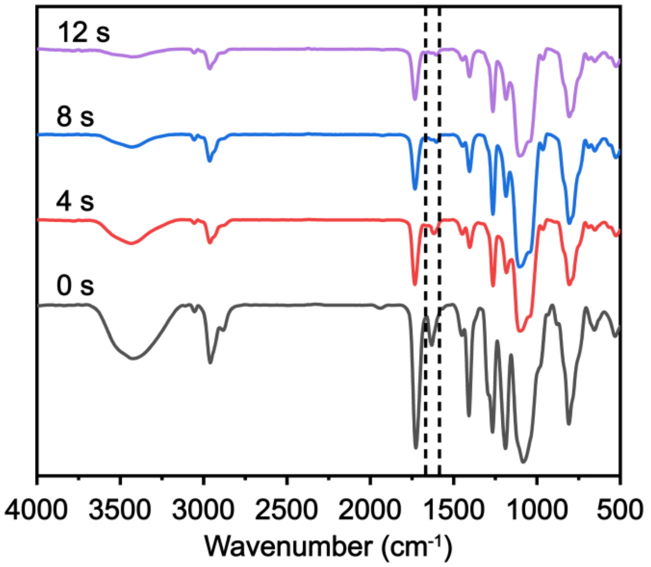

Fig. S3 The FT−IR spectra with different curing time

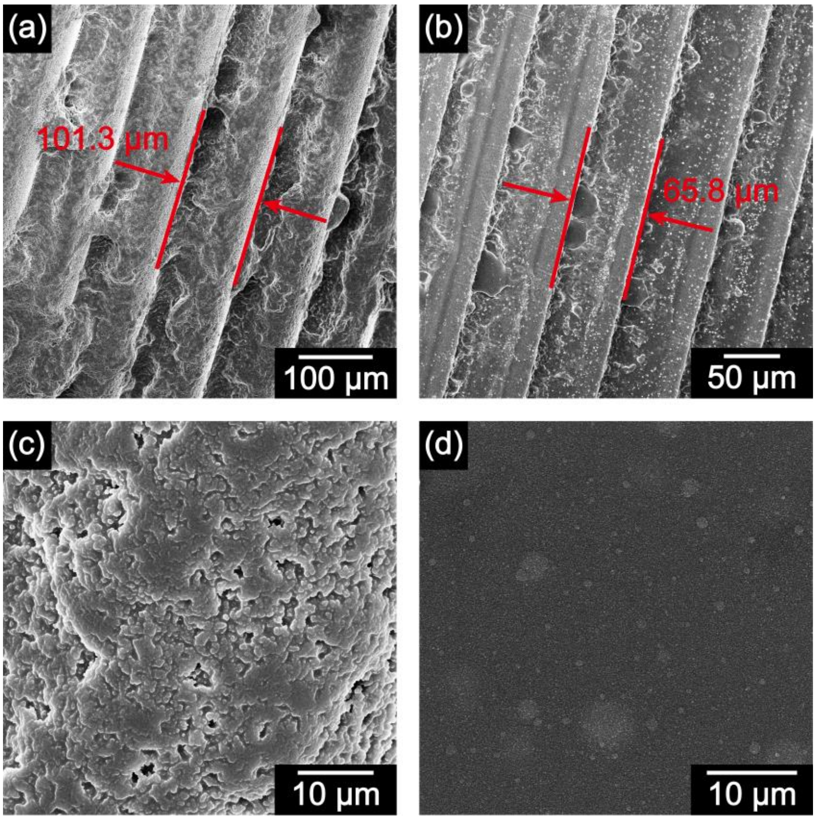

Fig. S4 (**a**) (**c**) SEM images of green body and (**b**) (**d**) corresponding ceramics pyrolyzed at 1200 °C in argon atmosphere

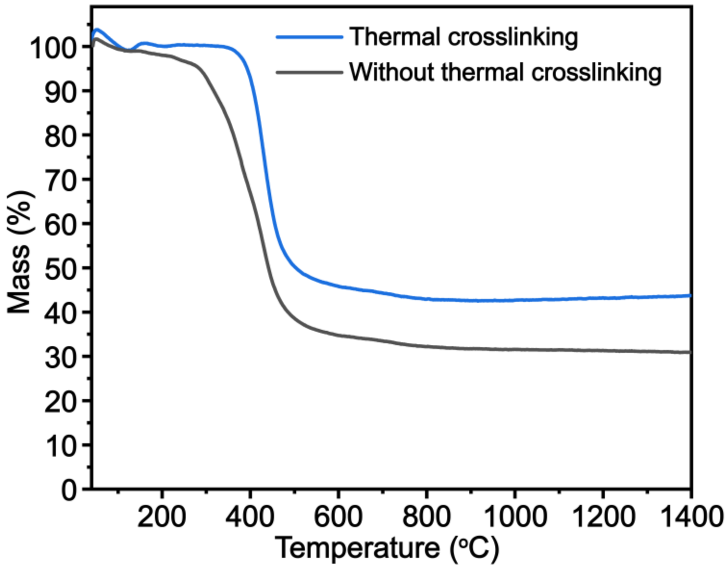

Fig. S5 TGA curves of thermal crosslinking and without thermal crosslinking 3D printing samples

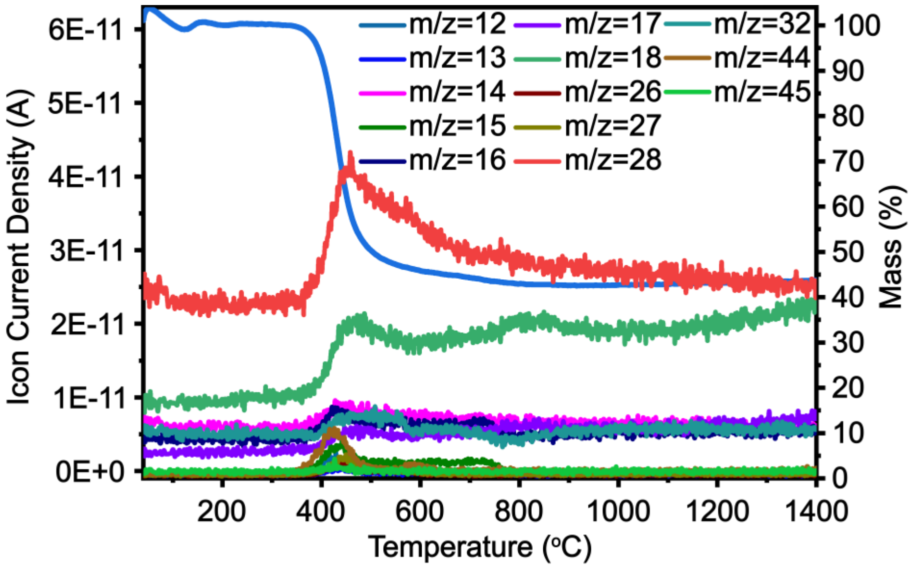

Fig. S6 TGA−MS spectra of green bodies with thermal crosslinking

As shown in **Fig. S5**, the slight mass loss of the no thermal crosslinking sample before 200 °C is mainly caused by the volatilization of residual small molecules and adsorbed water (less than 3%). From 200 °C, sample start to be degraded, while the thermal crosslinking sample begin to degrade at 350 °C. When the temperature increasing to 500 °C, the mass loss of thermal crosslinking sample is less than 55%, while the mass loss of the sample without thermal crosslinking is more than 65%. The thermal crosslinking of silicon vinyl provides a higher crosslinking density for the sample, thus reducing the mass loss during pyrolysis. From 500 °C to 1400 °C, both of the two samples barely show a mass loss (less than 5%). The thermal crosslinking sample has 43.74% ceramic yield. Furthermore, the TG−MS spectrometry in **Fig. S6** indicated the thermolysis was mainly accompanied by the decomposition products of hydrocarbons, CH_x^+ (x=0−3, m/z=12−15), CH_4 (m/z=16), $C_2H_x^+$ (x=2, 3, 4 and 6, m/z=26, 27, 28 and 30) as well as H_2 (m/z=2), OH^- (m/z=17), H_2O (m/z=18), O_2 (m/z=32), CO_2 (m/z=44) and other oligomer fragments (m/z=45). The evolution of CH_4, H_2, CH_x^+, $C_2H_x^+$ in the range from 300 °C to 500 °C was ascribed to decomposition of Si−CH_3 groups, −CH_2− groups and cleavage of C−C bonds. The O_2, CO and CO_2 were manly attributed to decomposition of C=O, −O− and the oxidation reaction. A small amount of −OH groups was due to the decomposition of Si−OH.

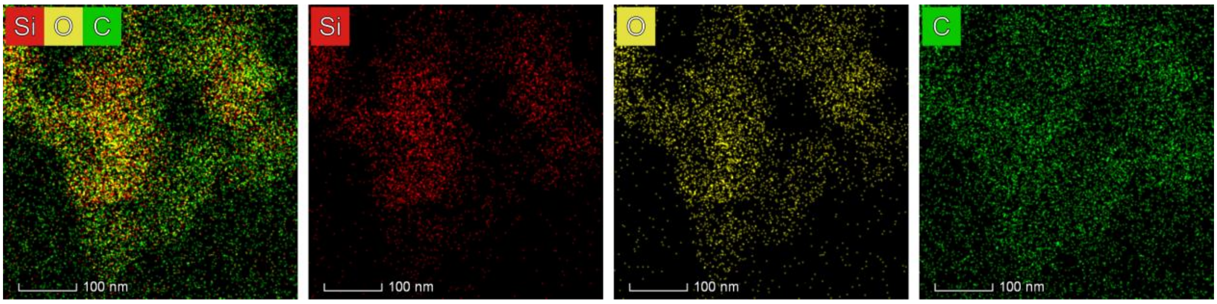

Fig. S7 The element mappings of Si, O, and C of the ceramic parts pyrolyzed at 1200 °C in argon atmosphere, respectively

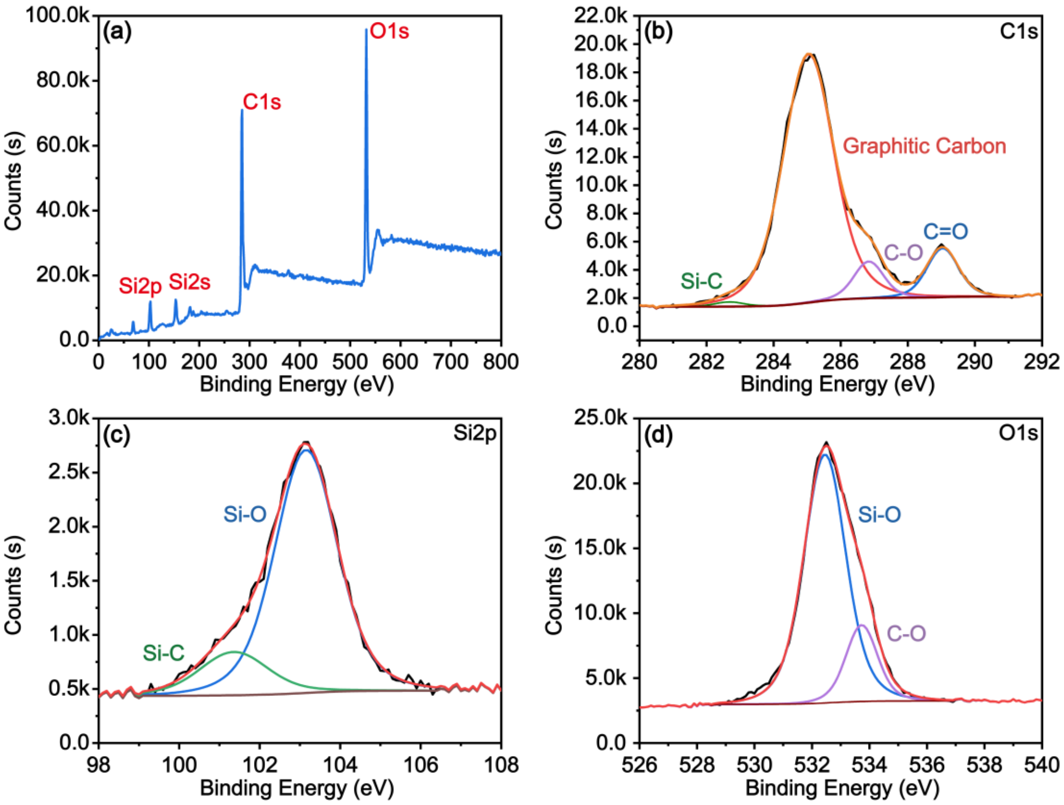

Fig. S8 XPS spectra of the ceramic parts pyrolyzed at 1200 °C in argon atmosphere. (**a**) XPS spectrum, (**b**) C 1s spectrum, (**c**) Si 2p spectrum, (**d**) O 1s spectrum

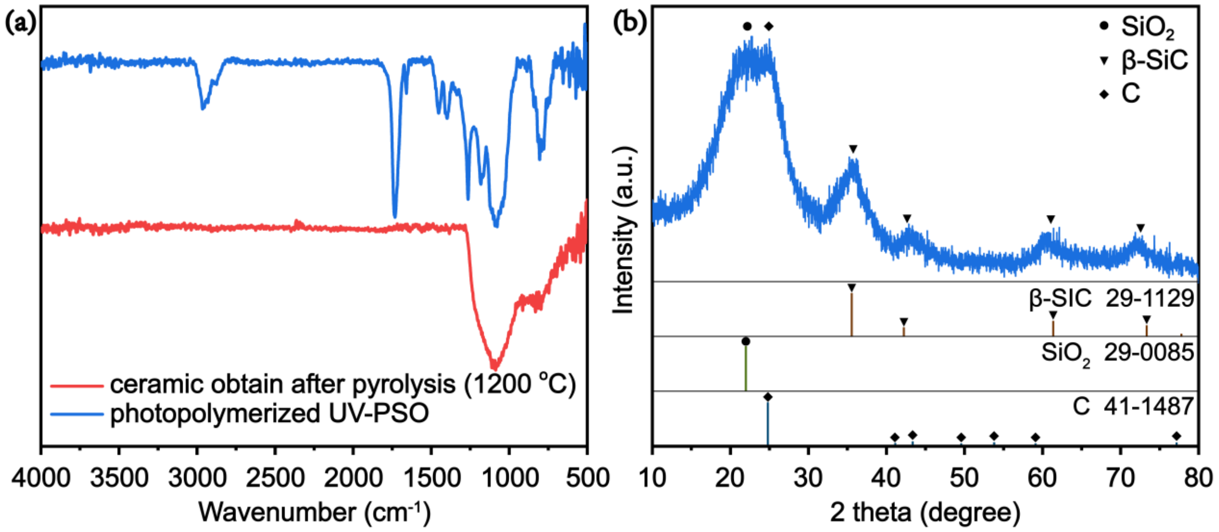

Fig. S9 (**a**) FT−IR spectra of the photo−polymerized UV−PSO before and after pyrolysis at 1200 ºC under argon atmosphere; (**b**) X−ray diffraction pattern recorded on the UV−PSO ceramic after pyrolysis at 1200 ºC under argon atmosphere

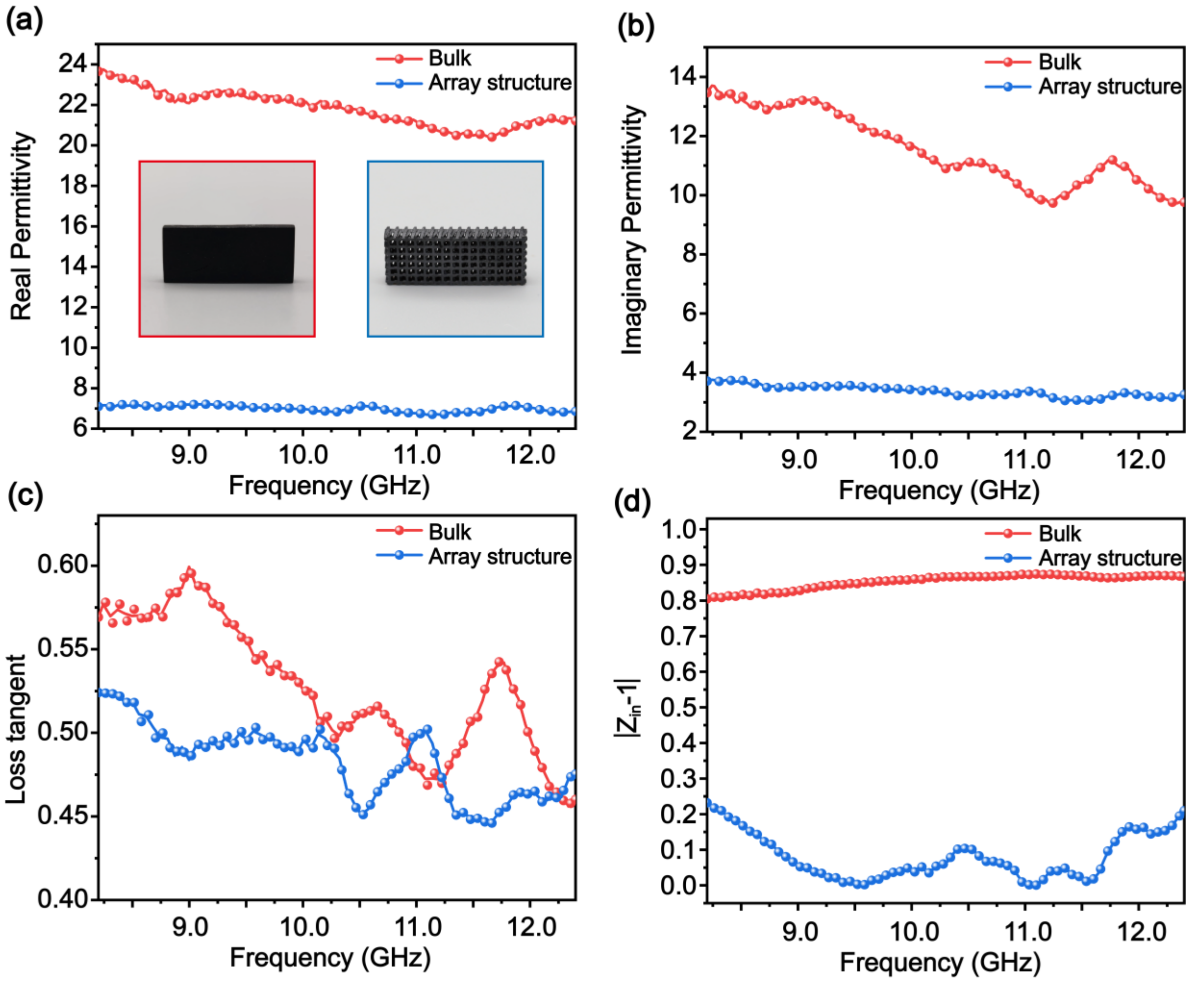

Fig. S10 The (**a**) real part, (**b**) imaginary part of permittivity and (**c**) loss tangent for 3D printed bulk ceramic sample and cross helix array structure ceramic sample; (**d**) modulus of $|Z_{in} - 1|$of samples at optimal thickness corresponding to RC_{min} in the frequency range 8.2−12.4 GHz

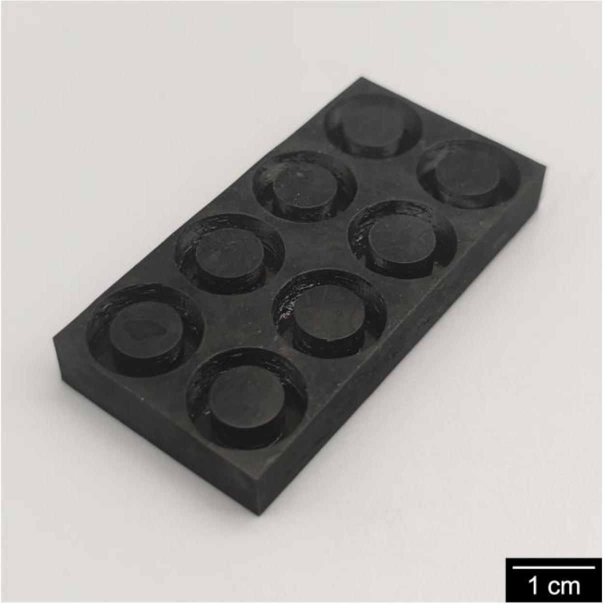

Fig. S11 Si−O−C ceramic metamaterial structure fabricated for EM wave absoption in X−Ku band

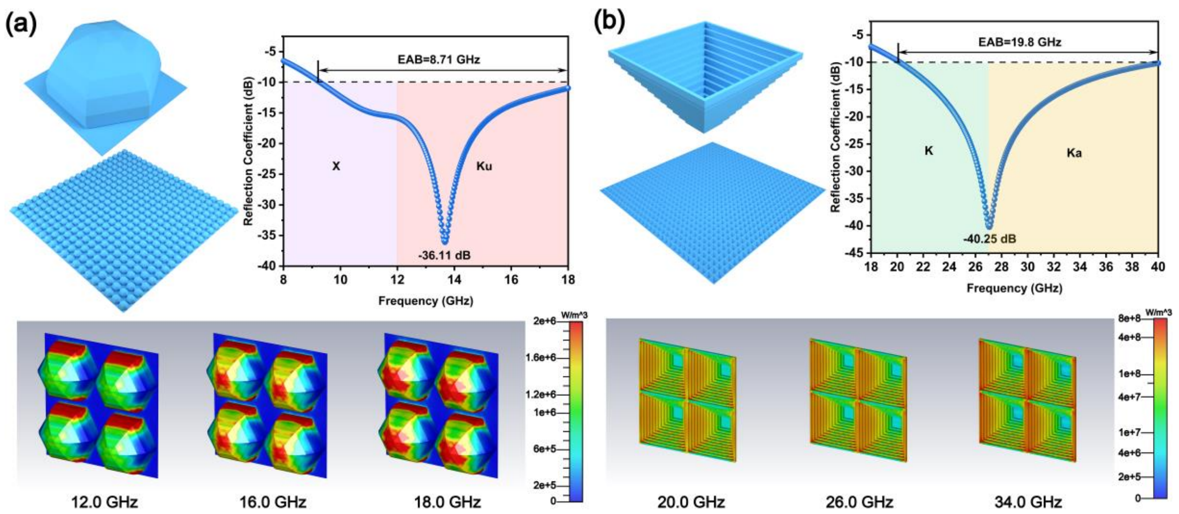

Fig. S12 Different metastructures for microwave absorbing in X, Ku, K, Ka bands and the corresponding RC data by simulations, based on the dielectric constant of the bulk sample
